# Mindfulness practice for protecting mental health during the COVID-19 pandemic

**DOI:** 10.1038/s41398-021-01459-8

**Published:** 2021-05-28

**Authors:** Julie Lei Zhu, Rasmus Schülke, Deniz Vatansever, Dayou Xi, Junjie Yan, Hanqing Zhao, Xiaohua Xie, Jianfeng Feng, Mark Yuting Chen, Barbara Jacquelyn Sahakian, Shouyan Wang

**Affiliations:** 1 Fanhai International School of Finance, Fudan University, Shanghai, China; 2 Institute of Science and Technology for Brain-Inspired Intelligence, Fudan University, Shanghai, China; 3 School of Economics, Fudan University, Shanghai, China; 4 Pure Awareness Research Institute, Shanghai, China; 5 Behavioural and Clinical Neurosciences Institute, Department of Psychiatry, University of Cambridge, Cambridge, United Kingdom

**Keywords:** Depression, Human behaviour

## Abstract

Emerging evidence shows that the coronavirus disease 2019 (COVID-19) pandemic is negatively affecting mental health around the globe. Interventions to alleviate the psychological impact of the pandemic are urgently needed. Whether mindfulness practice may protect against the harmful emotional effects of a pandemic crisis remains hitherto unknown. We investigated the influence of mindfulness training on mental health during the COVID-19 outbreak in China. We hypothesized that mindfulness practitioners might manifest less pandemic-related distress, depression, anxiety, and stress than non-practitioners and that more frequent practice would be associated with an improvement in mental health during the pandemic. Therefore, we assessed pandemic-related distress and symptoms of depression, anxiety, and stress, as well as the frequency of meditation practice at the peak of new infections (Feb 4–5; *N* = 673) and three weeks later (Feb 29–30; *N* = 521) in mindfulness practitioners via online questionnaires. Self-reported symptoms were also collected from non-practitioners at peak time only (*N* = 1550). We found lower scores of pandemic-related distress in mindfulness practitioners compared to non-practitioners. In general, older participants showed fewer symptoms of depression and anxiety. In younger practitioners, pandemic-related distress decreased from peak to follow-up. Importantly, increased mindfulness training during the preceding two weeks was associated with lower scores of depression and anxiety at both assessments. Likewise, practice frequency predicted individual improvement in scores of depression, anxiety, and stress at follow-up. Our results indicate that mindfulness meditation might be a viable low-cost intervention to mitigate the psychological impact of the COVID-19 crisis and future pandemics.

## Introduction

Mental disorders are a leading cause of disability with extensive socio-economic consequences. Conditions such as major depressive and anxiety disorders have a considerable impact on large portions of the population, with estimated global prevalences of 4.4% and 3.6%, respectively, in 2015^1^. Although a complex interaction of both biological and environmental influences gives rise to mental illness, a common risk factor that has long been identified is stress^2^. Stress triggered by major life events plays a pivotal role in the emergence of depressive symptoms, which are often proportional to the scale of the events and whether they involve interpersonal loss or social rejection^3^. Moreover, social isolation and loneliness are linked to higher levels of depressive symptoms^4–6^. Finally, global events like natural disasters, technological disasters, and terrorist acts severely increase the risk of depression across large populations^7^, necessitating specific and wide-scale interventions to alleviate their impact on mental health^8,9^.

A converging body of evidence suggests that the current viral outbreak may also act as a severe external stressor and have deleterious effects on public mental health^10^. The COVID-19 pandemic has given rise to fear concerning the disease’s health risks (for oneself and one’s loved ones)^11,12^ and its long-term economic consequences^12,13^. Furthermore, a recent review found that quarantine measures may increase symptoms of acute and post-traumatic stress, depression, and anger^14^.

A number of studies around the world have focused on assessing measures of stress, distress, depression, and anxiety across large population samples during the current COVID-19 outbreak. 54% of respondents in a survey conducted at the peak of the pandemic in China rated the psychological impact of the pandemic as moderate to severe, with 29% reporting moderate to severe symptoms of anxiety^15^. Two studies looked specifically at COVID-19 related distress and found mild to moderate distress in 29% and severe distress in 5.1% of Chinese respondents^16^, as well as 47% of mild to moderate distress and 14.1% of severe distress in Iranian respondents^17^. Likewise, an Italian survey conducted three weeks into the COVID-19 lockdown measures found high rates of symptoms of post-traumatic stress disorder, depression, anxiety, insomnia, and stress, especially in the case of COVID-related stressful life events, and discontinued working activity^18^. In another Italian study, students reported more depressive symptoms during lockdown compared to six months before^19^.

These studies highlight that mental health during pandemics is not only impacted in vulnerable groups like medical staff^20–22^ and patients with mental disorders^23^, but also in the general population. Therefore, protective factors and interventions to improve public mental health during global pandemics need to be identified and developed^24,25^. So far, factors such as continuing to work actively during the pandemic either in the usual workplace or home office^18,26^, physical exercise^26^ and limited media exposure^12,26^ have been linked to lower degrees of anxiety, depression, and stress and could thus be useful for mitigating the psychological impact of the current and future pandemic crises. However, the potential influence of daily practices directly aimed at improving mental health, such as mindfulness meditation, hitherto remains unknown.

Recently, mindfulness training interventions have emerged as a promising approach to foster mental health^27^. Originally based on ancient contemplative traditions, modern mindfulness interventions combine practices of relaxation and meditation into structured training, sometimes incorporating further elements of cognitive-behavioral therapy^28^. While specific implementations of mindfulness practices differ, these techniques usually involve the repeated practice of non-judgmental observation, in order to achieve mental stability and a state of mindfulness that is characterized by relaxed vigilance for distractions^27^. It has been shown in meta-analyses that mindfulness-based therapies are effective at reducing symptoms of depression and anxiety^29,30^. Mindfulness-based cognitive therapy has been validated as a clinical treatment for relapse prevention in recurrent depression^31^, was shown to be effective for treating current depressive symptoms^32^, and is being recommended for preventing depressive relapse by the United Kingdom’s National Institute for Health and Care Excellence^33^. In addition, mindfulness-based interventions reduce psychological stress^34^ by fostering stress resilience^35^ and are effective at reducing social anxiety disorder^36^. Mindfulness-based interventions are not only effective in clinical populations, but also reduce symptoms of distress, depression, anxiety, and stress in otherwise healthy individuals^37^. Mindfulness training may be especially beneficial in populations exposed to high levels of stress^38–40^. Previously, research centered on designing mindfulness interventions^41,42^ and elucidating its neural mechanisms^43,44^. Most studies on the efficacy of mindfulness treatments to alleviate symptoms of mental disorders have been conducted in research settings, and few investigations have focused on their impact in actual clinical practice or real-life environments^27^.

In this observational study, we investigated whether mindfulness practice under lockdown conditions during the COVID-19 pandemic might be associated with a change in mental health. First, we compared measures of mental health between mindfulness practitioners and non-practitioners at the peak of the COVID-19 pandemic in China, controlling for individual differences in age and sex. We hypothesized that mindfulness practitioners would show fewer symptoms of distress, depression, anxiety, and stress than non-practitioners. Moreover, we hypothesized a protective effect of mindfulness practice in practitioners, assessed a second time at a three-week follow-up. We postulated that increased mindfulness practice during the COVID-19 pandemic would be associated with positive changes in symptoms of pandemic-related distress, depression, anxiety, and stress, particularly in experienced practitioners.

## Methods

### Participants

Participants (Table 1) were recruited on the social media platform WeChat in China. Practitioners (assessed on Feb 4–5; *N* = 673, and Feb 29–30; *N* = 521) were directly recruited from a WeChat group of mindfulness practitioners practicing Pure Awareness mindfulness practices taught by the PARI (Pure Awareness Research Institute, see below). Non-practitioners (assessed on Feb 4–5; *N* = 1550) were recruited from the general population and reached via a broad advertising campaign on WeChat targeting no particular demographics. Experience of mindfulness practice in the practitioner group ranged between six months and two years. Participants were excluded from further analyses if they spent less than five/more than 30 min to complete the required questionnaires. Although the two groups did not differ significantly in gender (mean difference = 0.038; SE = 0.021; 95% CI = −0.004, 0.080; t(1317.255) = 1.782; *p* = 0.075), there was a significant age difference for each of the four categorical groups (25–30 y: mean difference = 0.090; SE = 0.012; 95% CI = 0.066, 0.114; t(1938.985) = 7.373; *p* < 0.001; 31–40 y: mean difference = 0.057; SE = 0.057; 95% CI = 0.013, 0.101; t(1305.391) = 2.531; *p* = 0.011; 41–50 y: mean difference = −0.099; SE = 0.022; 95% CI = −0.143, −0.055; t(1216.178) = −4.428; *p* < 0.001; 51–60 y: mean difference = −0.046; 95% CI = −0.076, −0.016; t(1100.141) = −3.020; *p* = 0.003; two-tailed tests, equal variances not assumed). These two demographic variables were included as predictors in subsequent statistical analyses.

**Table 1:** Sample characteristics.

		Non-practitioners at peak time	Practitioners at peak time	Practitioners at three-week assessment	Practitioners at peak time that were followed-up three weeks later
Total		1550	673	521	445
Age	25–30	218	34	28	22
	31–40	664	250	178	156
	41–50	500	284	229	196
	51–60	143	93	73	60
	>60	25	12	13	11
Sex	Female	1040	477	394	333
	Male	510	196	127	112
Lockdown status	Complete (all the time at home)	1124	423	325	285
	Partial (occasionally outside for work)	207	122	60	81
	None (working regularly)	219	128	136	79
Education	Junior high school education and lower	49	23	12	11
	Senior high school and equivalent	155	42	34	28
	Vocational education	324	115	88	71
	Undergraduate degree	685	280	220	191
	Graduate degree	337	213	167	144
Location	Hubei	70	16	12	11
	Beijing, Shanghai, Guangzhou, Shenzhen	704	305	169	208
	Other	776	352	340	226

### Study design

Pandemic-related distress, depression anxiety, and stress were assessed in non-practitioners (*N* = 1550) and practitioners (*N* = 673) at the peak of the COVID-19 pandemic in China (February 4–5, 2020). The same measures were assessed again at a three-week follow-up (*N* = 521; February 29–30, 2020) in an overlapping sample of mindfulness practitioners (*N* = 445 practitioners were assessed at both time points). All practitioners received instructions to practice meditation between the two assessment sessions and reported mindfulness practice frequency during the past two weeks. The study was approved by the local ethics committee (Fudan University). Informed consent was given at the beginning of the survey. Assessments were carried out via online questionnaires (SI-1 to SI-4). Distress was assessed using five questions related to the behavioral stress response to the COVID-19 pandemic (e.g., “I get nervous if someone nearby coughs or sneezes”, 1 [not nervous] to 7 [very nervous] Likert scale). Stress, anxiety, and depression were assessed with the 22-item Impact of Event Scale-Revised (IES-R)^45^ questionnaire, the 7-item Generalized Anxiety Disorder-7 questionnaire (GAD-7)^46^, and the 20-item Center for Epidemiologic Studies Depression Scale (CES-D)^47^, respectively, in Chinese^48^. The frequency of mindfulness practice was assessed with an 11-item questionnaire assessing the frequency of different mindfulness practices over the last two weeks (SI-5).

Practitioners were trained in mindfulness meditation by the PARI. Secular mindfulness practice as instructed by the PARI is designed to be applied in the workplace and domestic settings so as to be relevant to daily business and family life and does not include any spiritual or religious content. The training program practitioners underwent 6 to 24 months prior to the study teaches different exercises to increase mindfulness and reduce negatively valenced emotions like stress and anxiety. The practices trained are designed for autonomous practice after initial structured teaching courses (five 3-day courses to be taken over a 1-year period). The mindfulness practices trained here are mainly characterized by high degrees of meta-awareness and dereification in the phenomenological matrix of mindfulness practices proposed bz Lutz et al.^49^. Key features of the PARI mindfulness intervention are open-monitoring meditation, focused-attention meditation, and body scan meditation exercises. A specific exercise guide containing daily home mindfulness exercises for the period between February 8, 2020, and February 29, 2020, was developed and provided to the practitioners online. The guide included audio instructions for each exercise. Participants were asked to freely choose any exercises they wanted to practice. Based on former participation in structured courses, practitioners belonged to three subgroups. The courses taught increasingly advanced mindfulness meditation-related knowledge and skills and had to be taken in order; thus, practitioners that participated in beginner courses only were classified as beginners, and practitioners that took all courses were classified as advanced. All original information from both groups was anonymized with unique identification codes for further analyses before exporting the data from the online survey platform.

### Statistical analysis

Statistical analyses were performed using IBM SPSS Statistics (Version 26). The total score of each questionnaire (SI-1 to SI-5) was normalized to 0.0–1.0 (i.e., expressed as a proportion of the maximum score of the respective scale), in order to achieve similar distributions for statistical analysis. Measures of mental health and *Practice Frequency* were continuous variables, whereas the variables *Time*, *Group*, *Class*, *Sex*, and *Age* were categorical. Outliers (*z*-score >3 or <−3) were excluded before analysis. Since this was an observational study based on data collected during the height of the pandemic in China, no a priori power calculations could be performed. However, using G*Power we post-hoc computed the achieved statistical power^50^ for the ANOVAs and regression analyses. Because of the difference in sample sizes for the ANOVA of non-practitioners and practitioners at peak time, power calculations for the ANOVAs were conservatively based on a total sample size of *N* = 1346, twice the sample size of the smaller group. We used an alpha error probability of 0.05.

#### Group comparison

First, in order to investigate potential differences in pandemic-related distress, depression, anxiety, and stress between mindfulness practitioners (*N* = 1550) and non-practitioners (*N* = 673) at the peak of the pandemic, we conducted four ANOVAs, including the main effects *Group* (non-practitioner, beginner, intermediate, advanced), *Age* (25–30, 31–40, 41–50, 51–60, ≥61 years) and *Sex* (male, female), as well as their two-way interactions in the model. We were mainly interested in the main effect of *Group*, as well as the interactions including the factor *Group*. Since exploratively including three-way interactions in the models did not result in any significant effects and did not improve model fit, assessed using adjusted *R*^2^, three-way interactions were not included in the final models. For the four ANOVAs, Bonferroni correction was applied by adjusting the significance level for main effects and interactions: Statistical significance was accepted at *p* < 0.0125. Pairwise comparisons were performed to follow-up significant main effects. Here, FDR adjustment was applied.

#### Change within practitioners

To probe whether the mental health of mindfulness practitioners changed during the initial phase of the COVID-19 pandemic, we compared pandemic-related distress, depression, anxiety, and stress at peak time (*N* = 673) and at three-week follow-up (*N* = 521; for *N* = 445 practitioners data were available at both time points) using hierarchical linear models (HLMs). We included the categorical variables *Time* (peak, three-week follow-up), *Experience* (beginner, intermediate, advanced), *Age* (25–30, 31–40, 41–50, 51–60, ≥61 years), and *Sex* (male, female) and the continuous variable *Practice Frequency* as main effects in the model, as well as all two-way interactions. A diagonal covariance structure was selected for the repeated measures. Since adding the respective three-, four-way, and five-way interactions did not improve model fit, as assessed with Akaike information criteria, and did not result in significant effects, these were not retained in the final model. We were primarily interested in significant main effects or interactions of *Time*, *Practice Frequency*, and *Experience*. Bonferroni correction was applied to correct for running four HLMs by adjusting the significance level for main effects and interactions: Statistical significance was accepted at *p* < 0.0125. Pairwise comparisons were performed to follow-up the significant interaction and main effects. For pairwise comparisons, FDR adjustment was applied.

#### Practice effect

To test our main hypothesis of potential mindfulness practice effects on mental health during the pandemic, change in individual symptom scores of mindfulness practitioners (*N* = 445) was regressed on *Practice Frequency* during the last two weeks while controlling for *Age* (25–30, 31–40, 41–50, 51–60, ≥61 years), *Sex* (male, female) and baseline symptoms. The age categories were recoded into four dummy variables. The change was computed as the score of distress/depression/anxiety/stress at peak time minus the same score at three-week follow-up, for each practitioner. Thus, positive changes indicated improvement. We hypothesized a dose-response relationship between *Practice Frequency* and improvement in measures of mental health during the COVID-19 pandemic. Bonferroni correction was applied by adjusting the threshold for accepting statistical significance for the overall models and the individual regression coefficients to *p* < 0.0125. The regression analyses were then repeated after splitting the data into beginner, intermediate, and advanced practitioner subgroups. Here, FDR adjustment (12 follow-up regression analyses) was performed (for both the significance of the models and the regression coefficients). Finally, to assess the relationship between practice frequency and symptoms at baseline, we calculated the Pearson correlation coefficient.

## Results

### Group comparison

**Fig. 1:**
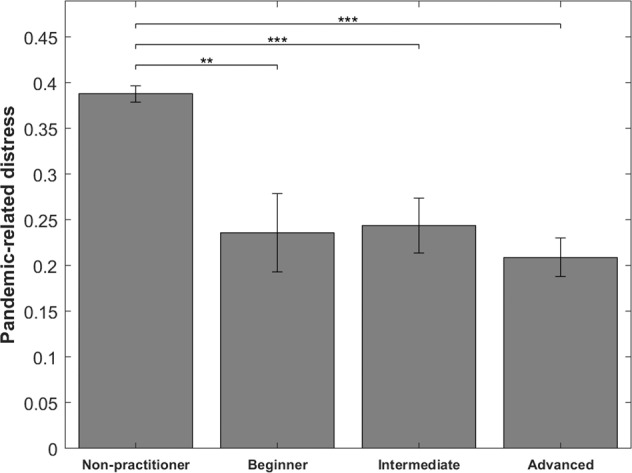
Pandemic-related distress at peak. Mindfulness practitioners manifested less pandemic-related distress than non-practitioners. Shown are the estimated marginal means of self-reported pandemic-related distress at peak time for practitioners (beginner, intermediate, advanced) and non-practitioners. **p  < 0.01, ***p < 0.001. Error bars indicate the standard error.

First, we compared mental health between mindfulness practitioners and non-practitioners at the peak of the COVID-19 outbreak in China, running four separate ANOVAs (supplementary table 1, supplementary table 2 for descriptive statistics). There was a significant main effect of *Group* for scores of pandemic-related distress (F(3, 2195) = 27.487; *p* < 0.001; *η*_p_^2^ = 0.036; achieved power = 1; Fig. 1). Pairwise comparisons indicated that non-practitioners differed significantly from practitioners at beginner (mean difference = .151; SE = 0.044; t(1796) = 3.432; 95% CI = 0.065, 0.238; *p*_FDR-adjusted_ = 0.001; mean difference in raw scores = 4.53), intermediate (mean difference = 0.144; SE = 0.031; t(1690) = 4.645; 95% CI = 0.082, 0.205; *p*_FDR-adjusted_ < 0.001; mean difference in raw scores = 4.32) and advanced (mean difference = 0.179; SE = 0.023; t(1831) = 7.783; 95% CI = 0.135, 0.223; *p*_FDR-adjusted_ < 0.001; mean difference in raw scores = 5.37) experience levels, reporting less pandemic-related distress. There were no significant differences between subgroups of practitioners and no significant effects of *Group* for scores of depression, anxiety, and stress. However, the main effects of *Age* for scores of depression (F(4, 2195) = 5.912; *p* < 0.001; *η*_p_^2^ = 0.011; achieved power = 0.867) and anxiety (F(4, 2195) = 6.455; *p* < 0.001; *η*_p_^2^ = 0.012; achieved power = 0.898; Supplementary Fig. 1) were significant. Regarding depressive symptoms, pairwise comparisons showed that subjects aged 25–30 y reported significantly more symptoms than subjects aged 41–50 y (mean difference = 0.044; SE = 0.019; t(1834) = 2.316; 95% CI = 0.006, 0.081; *p*_FDR-adjusted_ = 0.043; mean difference in raw scores = 2.64) and >60 y (mean difference = 0.106; SE = 0.042; t(387) = 2.524; 95% CI = 0.023, 0.188; *p*_FDR-adjusted_ = 0.031; mean difference in raw scores = 6.36). Likewise, subjects aged 31–40 y also showed significantly more symptoms than older subjects aged 41–50 y (mean difference = 0.036; SE = 0.009; t(2247) = 4.0; 95% CI = 0.018, 0.054; *p*_FDR-adjusted_ = 0.001; mean difference in raw scores = 2.16), 51–60 y (mean difference = 0.035; SE = 0.014; t(1513) = 2.5; 95% CI = 0.008, 0.062; *p*_FDR-adjusted_ = 0.037; mean difference in raw scores = 2.10) and >60 y (mean difference = 0.098; SE = 0.031; t(1250) = 2.513; 95% CI = 0.022, 0.174; *p*_FDR-adjusted_ = 0.031; mean difference in raw scores = 5.88). Similarly, with regard to anxiety scores, pairwise comparisons showed that participants aged 25–30 y reported significantly more anxiety symptoms than subjects over 60 y (mean difference = 0.119; SE = 0.049; t(387) = 2.429; 95% CI = 0.022, 0.215; *p*_FDR-adjusted_ = 0.041; mean difference in raw scores = 3.33), and subjects aged 31–40 y also manifested more symptoms than subjects aged 41–50 y (mean difference = 0.045; SE = 0.011; t(2247) = 4.091; 95% CI = 0.023, 0.066; *p*_FDR-adjusted_ < 0.001; mean difference in raw scores = 1.26), 51–60 y (mean difference = 0.049; SE = 0.016; t(1513) = 3.063; 95% CI = 0.018, 0.081; *p*_FDR-adjusted_ = 0.010; mean difference in raw scores = 1.37) and >60 y (mean difference = 0.121; SE = 0.045; t(1250) = 2.689; 95% CI = 0.032, 0.210; *p*_FDR-adjusted_ = 0.026; mean difference in raw scores = 3.39). Thus, younger participants below the age of 40 reported more symptoms of depression and anxiety than older participants. No other main effects or interactions were significant at the Bonferroni corrected significance threshold of *p* < 0.0125.

### Change in practitioners

**Fig. 2:**
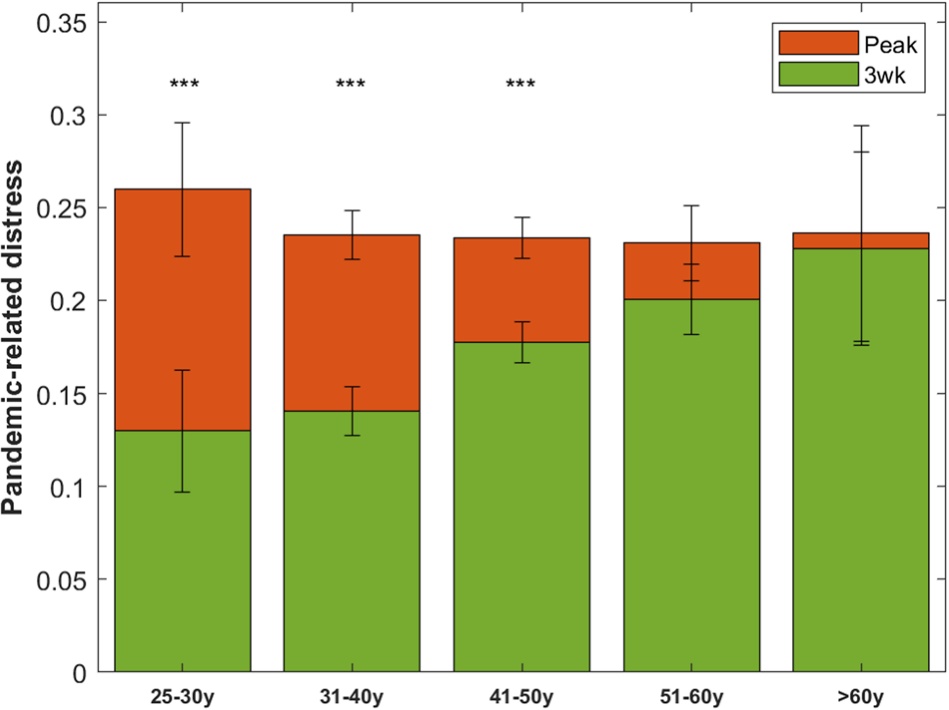
Age-dependent decrease of pandemic-related distress. Pandemic-related distress decreased from peak to follow-up in younger mindfulness practitioners. Shown are the estimated marginal means of self-reported pandemic-related distress in practitioners at peak and three-week follow-up in the different age groups. ***p < 0.001. Error bars indicate the standard error. *y* = years.

To investigate changes in the mental health status of mindfulness practitioners during the COVID-19 pandemic in China, we performed hierarchical linear modeling for scores of pandemic-related distress, depression, anxiety, and stress (supplementary table 3, supplementary table 2 for descriptive statistics). For pandemic-related distress, we found a significant interaction of *Time* *×* *Age* (F(4, 546.106 = 4.007; *p* = 0.003; Fig. 2). Pairwise comparisons showed that pandemic-related distress decreased from peak to follow-up in younger practitioners. Specifically, pandemic-related distress decreased significantly in the age groups 25–30 y (mean difference = 0.130; SE = 0.031; t(595.079) = 4.194; 95% CI = 0.069, 0.190; *p*_FDR-adjusted_ < 0.001; mean difference in raw scores = 3.90), 31–40 y (mean difference = 0.0.095; SE = 0.013; t(579.642) = 7.308; 95% CI = 0.070, 0.120; *p*_FDR-adjusted_ < 0.001; mean difference in raw scores = 2.85) and 41–50 y (mean difference = 0.056; SE = 0.011; t(554.951) = 5.091; 95% CI = 0.034, 0.078; *p*_FDR-adjusted_ < 0.001; mean difference in raw scores = 1.68).

For depressive symptoms, we found a significant main effect of *Practice Frequency* (F(1, 1060.453) = 12.527; *p* < 0.001). There was a significant negative Pearson correlation between *Practice Frequency* and depressive symptoms (*r* = −0.286, *p* < 0.001), the more frequent practice was associated with lower reported symptoms. Furthermore, we found a significant main effect of *Time* (F(1, 546.766) = 20.353; *p* < 0.001), due to practitioners reporting more depressive symptoms at follow-up (mean difference = −0.042; SE = 0.010; t(488.304) = −4.372; 95% CI = −0.061, −0.023; *p*_FDR-adjusted_ < 0.001; mean difference in raw scores = −2.52).

For symptoms of anxiety, we again found a significant main effect of *Practice Frequency* (F(1, 921.902) = 15.103; *p* < 0.001). There was a significant negative Pearson correlation between practice frequency and anxiety symptoms (*r* = −0.263, *p* < 0.001). We also found a significant main effect of *Age* (F(4, 948.160) = 3.370; *p* = 0.009). Younger practitioners aged 25–30 y showed significantly more symptoms of anxiety than practitioners aged 41–50 y (mean difference = 0.076; SE = 0.026; t(663.157) = 2.923; 95% CI = 0.025, 0.127; *p*_FDR-adjusted_ = 0.018; mean difference in raw scores = 2.13) and 51–60 y (mean difference = 0.074; SE = 0.028; t(678.537) = 2.643; 95% CI = 0.018, 0.130; *p*_FDR-adjusted_ = 0.033; mean difference in raw scores = 2.07). Similarly, practitioners aged 31–40 y showed more anxiety than practitioners in the age group 41–50 y (mean difference = 0.038; SE = 0.012; t(746.099) = 3.167; 95% CI = 0.016, 0.061; *p*_FDR-adjusted_ = 0.009; mean difference in raw scores = 1.06).

No other main effects or interactions resulted in significant effects at the Bonferroni corrected significance threshold of *p* < 0.0125.

### Practice effect

**Fig. 3:**
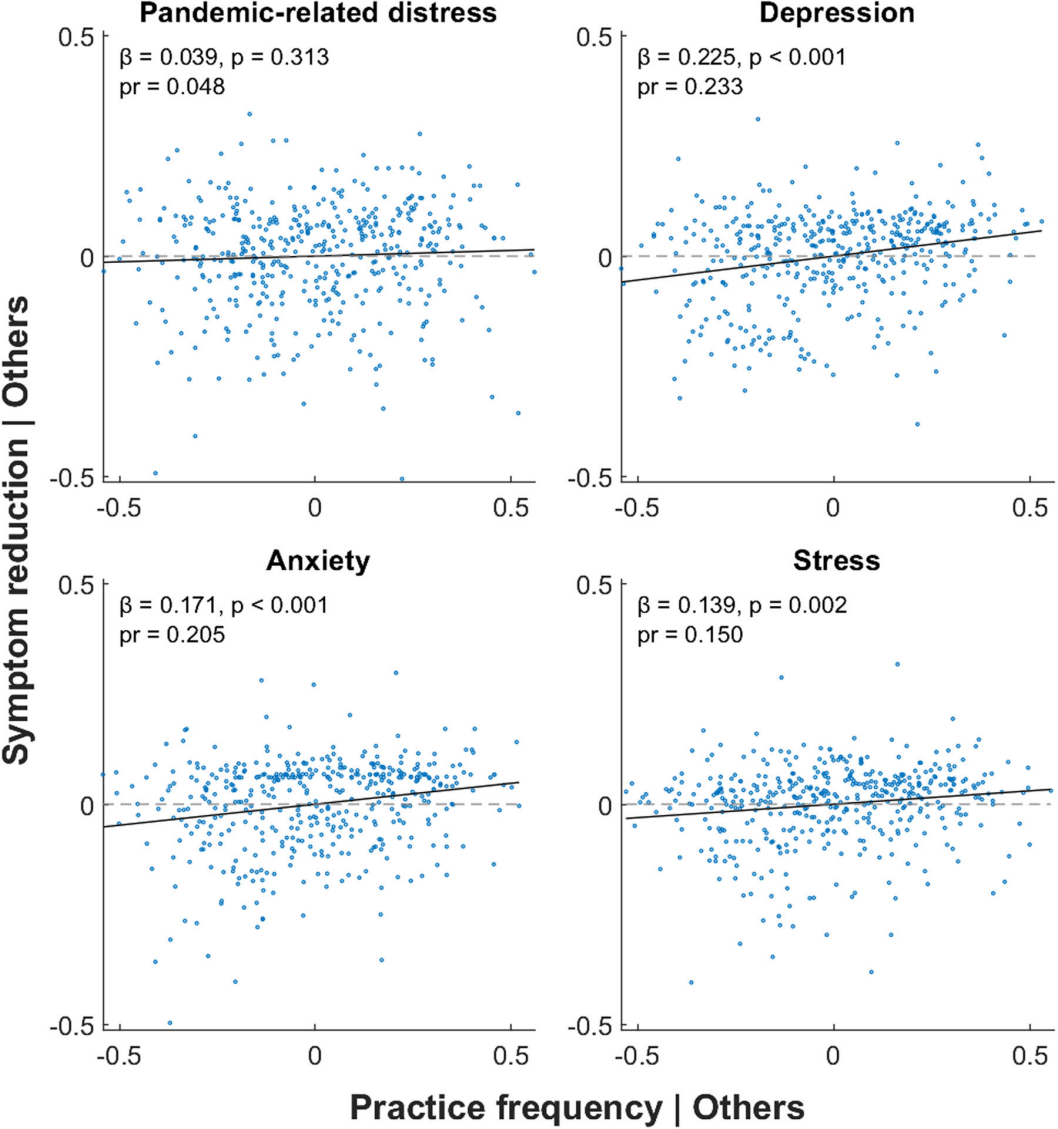
Practice-dependent symptom improvement. Partial regression plots showing the improvement in self-reported symptoms of depression (normalized CES-D score at peak − normalized CES-D score at three-week follow-up), anxiety (normalized GAD-7 score at peak − normalized GAD-7 score at three-week follow-up), and stress (normalized IES-R score at peak − normalized IES-R score at three-week follow-up) with increased frequency of mindfulness practice when controlling for other variables (age, sex, and symptoms at baseline). β = beta coefficient, pr = partial correlation.

To further test whether individual changes in symptom scores were related to practice frequency, the change in symptom scores (available at both time points for *N* = 445 practitioners) was regressed on practice frequency during the last two weeks (supplementary table 4). *Practice Frequency* significantly predicted improvement for symptoms of depression (ANOVA: F(7437) = 15.586; *p* < 0.001; *R*^2^ = 0.200; Coefficient: *B* = 0.108; SE_B_ = 0.022; CI = 0.066, 0.151; *β* = 0.225; t(437) = 5.003; *p* < 0.001; *pR*^2^ = 0.054; achieved power = 0.999), anxiety (ANOVA: F(7,437) = 40.825; *p* < 0.001; *R*^2^ = 0.395; Coefficient: *B* = 0.095; SE_B_ = 0.022; CI = 0.052, 0.137; *β* = 0.171; t(437) = 4.374; *p* < 0.001; *pR*^2^ = 0.042; achieved power = 0.993) and stress (ANOVA: F(7437) = 18.024; *p* < 0.001; *R*^2^ = 0.224; Coefficient: *B* = 0.061; SE_B_ = 0.019; CI = 0.023, 0.098; *β* = 0.139; t(437) = 3.176; *p* = 0.002; *pR*^2^ = 0.023; achieved power = 0.892) when including *Age*, *Sex* and the respective baseline scores of depression/anxiety/stress as control variables; the more frequent practice was associated with symptom reduction (Fig. 3). For distress, we did not find an effect of *Practice Frequency*. Looking at practitioners with different levels of mindfulness practice experience (supplementary table 5), in advanced practitioners, the effect of *Practice Frequency* was significant for the improvement of depressive symptoms (ANOVA: F(7202) = 11.050; *p*_FDR-adjusted_ < 0.001; *R*^2^ = 0.277; Coefficient: *B* = 0.105; SE_B_ = 0.032; CI = 0.043, 0.168; *β* = 0.211; t(202) = 3.316; *p*_FDR-adjusted_ = 0.006; *pR*^2^ = 0.052) and improvement of anxiety symptoms (ANOVA: F(7202) = 16.763; *p*_FDR-adjusted_ < 0.001; *R*^2^ = 0.367; Coefficient: *B* = 0.108; SE_B_ = 0.030; 95% CI = 0.049, 0.168; *β* = 0.211; t(202) = 3.596; *p*_FDR-adjusted_ = 0.005; *pR*^2^ = 0.060); more practice was associated with symptom reduction. For depression, the effect of *Practice Frequency* was also significant in intermediate practitioners (ANOVA: F(7, 81) = 4.721; *p*_FDR-adjusted_ < 0.001; *R*^2^ = 0.290; Coefficient: *B* = 0.122; SE_B_ = 0.045; 95% CI = 0.033, 0.212; *β* = 0.272; t(81) = 2.724; *p*_FDR-adjusted_ = 0.032; *pR*^2^ = 0.084).

Regarding the relationship between *Practice Frequency* and baseline symptoms, we found significant negative associations between *Practice Frequency* and baseline scores of pandemic-related distress (*r* = −0.189; *p* < 0.001), depression (*r* = −0.239; *p* < 0.001), anxiety (−0.259; *p* < 0.001) and stress (*r* = −0.197; *p* < 0.001).

## Discussion

Since emerging evidence suggests that the COVID-19 pandemic and the counter-measures it engendered have a considerable negative impact on mental health^12,15–18^, it is crucial to identify scalable, low-cost interventions that can safeguard public mental health during this and future pandemic crises^9,25^. In our observational study, we investigated the relationship of mindfulness practice and pandemic-related distress, depressive symptoms, anxiety symptoms, and stress, in a large group of mindfulness practitioners, surveyed at the peak of new COVID-19 infections in China and three weeks later. Symptom scores of practitioners at peak time were also compared to a group of non-practitioners, surveyed at peak time only.

### Group comparison

The level of pandemic-related distress reported at peak time was significantly lower in practitioners, compared to non-practitioners, when controlling for possible confounding effects of age and sex. We found no differences in symptoms of depression, anxiety, and stress between both groups. These findings might be explained by the fact that pandemic-related distress could be more sensitive to specific emotional/behavioral pandemic-related alterations (e.g., getting nervous if someone nearby coughs or sneezes). Such symptoms might be more amenable to change (even after limited amounts of mindfulness practice) than clinical and more trait-like characteristics like symptoms of depression, anxiety, or stress. We also found that older participants manifested generally fewer symptoms of depression and anxiety, compared to younger participants, regardless of group. This finding agrees with former studies reporting age-related effects for depression and anxiety^50,51^.

### Change in practitioners

In line with our hypothesis of protective psychological effects of mindfulness practice during the COVID-19 pandemic, we found a reduction in pandemic-related distress at follow-up in younger practitioners. We did not find significant differences in anxiety or stress between the peak and three-week follow-up surveys. It is possible that a putative negative psychological impact of the pandemic has been protected against by a positive influence of mindfulness meditation practice. In support of this interpretation, considerable evidence has demonstrated that mindfulness meditation may reduce anxiety, stress, and depressive symptoms and that the COVID-19 pandemic has generally increased such symptoms^9^. Indeed, practice frequency correlated negatively with symptoms of depression and anxiety. However, we also found an increase in depressive symptoms from peak to follow-up. This increase could—in part—be due to the strict quarantine measures during the time of this study^14,19^.

### Practice effect

The regression of symptom improvement on practice frequency before follow-up showed a dose-dependent reduction in symptoms of anxiety, depression, and stress. Practitioners who practiced more frequently during the critical phase of the COVID-19 pandemic in China had a better mental health status at follow-up than those practicing less frequently. This association was most robust for depression (partial correlation squared = 0.054). Analyzing this association in each of the three subgroups separately, we found the strongest association in advanced practitioners (for both depression and anxiety). Thus, the effectiveness of autonomous mindfulness practice may depend critically on an appropriate amount of previous structured training. Interestingly, even though more frequent mindfulness practice was associated with fewer symptoms of depression, anxiety, and stress in practitioners, practitioners did not differ significantly from non-practitioners on these variables at peak. The relative weakness of the associations between practice frequency and symptom improvement might explain why the between-group comparison did not result in significant effects. Conversely, despite the group difference regarding pandemic-related distress, we did not find an association between practice frequency and pandemic-related distress in practitioners. The group difference in pandemic-related distress might thus be due to other factors independent of mindfulness practice frequency that could be related to mindfulness practice (e.g., better emotional control resulting from long-term practice) but could also be independent of mindfulness practice altogether (i.e., confounding socio-demographic variables).

### Comparison with other findings and potential mechanisms of mindfulness practice

Regular mindfulness practice is accompanied by structural and functional changes in brain regions involved in the regulation of emotion, attention, and self-awareness^43,52^. Consequently, mindfulness-based interventions have been increasingly studied as a treatment tool for psychiatric conditions such as depression and anxiety^27,29,53^. Our findings are in line with previous meta-analyses showing that mindfulness-based interventions are useful treatments for reducing distress, anxiety, depression, and stress^29^ that are not only effective in clinical populations but also improve mental health in healthy individuals^37^. The dose-dependent reduction in symptoms of depression, anxiety, and stress concords well with former studies demonstrating that mindfulness practice can improve stress resilience^35,54^ and results in an enhanced ability to find meaning in adverse events^55^, like the COVID-19 pandemic.

Emotion regulation is known to be dysfunctional across many mental disorders^56,57^, such as depression^58^. Dysfunctional emotion regulation during a time of crisis will likely have a particularly detrimental effect on one’s mental health status. Mindfulness practice strengthens the ability to consciously notice emotional states and improves their regulation^43,59–62^. The emotion regulation strategies relevant for mindfulness practice can be differentiated into top-down strategies (e.g., affect labeling) and bottom-up strategies (e.g., sensory-perception)^63^. It has been suggested that top-down emotion regulation strategies may be more relevant in short-term mindfulness practitioners, while bottom-up strategies could play a greater role in long-term practitioners^64^, such as the advanced practitioners in our study. Since emotional self-regulation is at the core of resilience, an increase in resilience due to mindfulness practice could protect against stress and anxiety during the pandemic. Increases in stress resilience due to mindfulness training have also been reported for other contexts with high levels of stress^38–40^. Recently, resilience has been shown to protect against COVID-19 related distress and was linked to lower rates of anxiety and depression^11^. Further research should investigate the relationship between mindfulness practice, emotion regulation, resilience, and mental health status.

Besides, other mechanisms play important roles in the effects of mindfulness-based interventions. A systematic review of mindfulness-based cognitive therapy (MBCT) in the treatment of recurrent major depressive disorder found that alterations in mindfulness, ruminations, worries, and meta-awareness were associated with, predicted, or mediated the effects of MBCT interventions^65^. In particular, ruminations—excessive, repetitive, and uncontrolled negatively valenced thoughts—are likely to be increased during a time of crisis like the COVID-19 pandemic and have been shown to decrease under MBCT in a randomized controlled trial^66^. Reductions in ruminations, worrying, and unconstructive repetitive thoughts in general^67^ due to mindfulness meditation training may partly explain the reductions in scores of depression, anxiety, and stress in this study. A decrease in mind-wandering and higher meta-awareness of distraction^68^ and non-specific aspects of mindfulness practice such as increased self-efficacy may also play a role. Our findings highlight that an appropriate amount of guided mindfulness training is crucial for self-administrated mindfulness practice to be effective under real-life conditions. Differential effects of different levels of mindfulness training experience have also been investigated in recent fMRI studies. For example, the total amount of retreat meditation practice correlated with a reduction of right amygdala activation for negative pictures in experienced practitioners having accumulated about 9000 h of lifetime meditation practice, while no reduction in amygdala sensitivity to negative stimuli was seen after a single 8-week mindfulness-based stress reduction (MBSR) program^69^. In contrast, another neuroimaging study found that changes in hippocampal-cortical connectivity occurred even after a single 8-week MBSR intervention^35^. These investigations illustrate that changes in brain network activity underlying beneficial effects of mindfulness practice accrue over time in a complex manner, in agreement with greater benefit in advanced practitioners in our study. Unfortunately, subjects participating in MBSR/MBCT interventions often do not carry out the recommended amount of home practice^70^. In our investigation, practitioners received web-based instructions to encourage mindfulness training during the pandemic. In the future, internet-based and smartphone-based mindfulness interventions could be a low-cost option to make mindfulness-based treatment available to large populations^9,71^. This could also overcome the challenge of appropriately training mindfulness meditation teachers, identified as one of the barriers hindering the translation of mindfulness research into clinical practice^72^.

### Limitations

Causal interpretations of our data are limited by the observational nature of our study. Selection bias may have contributed to the difference between non-practitioners and practitioners. It is possible that practitioners and non-practitioners differed in unmeasured characteristics such as environmental and socioeconomic factors or personality/cognitive styles, which might explain why practitioners experienced less pandemic-related distress. Furthermore, the interpretability of the comparison between measures at peak time and three-week follow-up in practitioners would have been improved if the sample of non-practitioners could have been followed-up for a second assessment as well since mental states might have worsened during the pandemic in non-practitioners. Finally, it needs to be noted that the magnitude of mindfulness practice effects in this study is relatively small. However, it is encouraging that a positive association between frequency of practice and improvement in symptoms of depression, anxiety, and stress could be found, given that we looked at only three weeks of unsupervised, autonomous practice (thus, one expects a considerably smaller effect than for classic 8-week MBSR programs involving considerable didactic instruction, frequent group sessions and a daylong retreat)^27^. Further experimental studies should corroborate our findings and elucidate whether more prolonged or more intensive mindfulness practice may elicit stronger protective effects during the pandemic.

## Conclusion

Our observational study demonstrates that practitioners of mindfulness meditation manifested less pandemic-related distress than non-practitioners during the COVID-19 pandemic in China. Importantly, practice frequency was associated with improvements in symptoms of depression, anxiety, and stress, especially in experienced practitioners. Further interventional studies should corroborate our results. If implemented effectively, mindfulness-based interventions might be a low-cost option to safeguard public mental health at times of crisis like the current COVID-19 pandemic.

## Supplementary information

Supplementary information

Supplementary table 1

Supplementary table 2

Supplementary table 3

Supplementary table 4

Supplementary table 5

Supplementary figure 1
