# Supplementary material for: Mindfulness practice for protecting mental health during the COVID-19 pandemic
Source: Transl Psychiatry. 2021 May 28;11:329. doi: 10.1038/s41398-021-01459-8 (PMC8160402; doi:10.1038/s41398-021-01459-8)
Supplement: Supplementary file 1 — Supplementary information [file 41398_2021_1459_MOESM1_ESM.docx]

**SI-1 Questionnaire for Assessment of Distress**

1. I get nervous if someone nearby coughs or sneezes. (1-not nervous, 7-very nervous）
2. I can’t help checking on news regarding the pandemic. (1-strongly disagree. 7-strongly agree)
3. I frequently check my body temperature, the oxygen content in my blood, make use of online screening tests, and check whether myself or my family members have signs of disease. (1-strongly disagree. 7-strongly agree)
4. I have been forwarding news and reports regarding the pandemic (including articles from various WeChat media channels) and am very enthusiastic about taking part in the relevant discussions. (1-strongly disagree. 7-strongly agree)
5. I am worried that this pandemic will cause economic depression, bankruptcy of enterprises or unemployment. (1-strongly disagree. 7-strongly agree)

**SI-2 Questionnaire for Assessment of Stress**

Stress Assessment (Impact of Event Scale-Revised (IES-R) questionnaire in Chinese) (1: no symptoms, 2: mild, 3: moderate, 4: severe, 5: very severe)

1. Any reminder brought back feelings about it.
2. I had trouble staying asleep.
3. Other things kept making me think about it.
4. I felt irritable and angry.
5. I avoided letting myself get upset when I thought about it or was reminded of it.
6. I thought about it when I didn’t mean to.
7. I felt as if it hadn’t happened or wasn’t real.
8. I stayed away from reminders of it.
9. Pictures about it popped into my mind.
10. I was jumpy and easily startled.
11. I tried not to think about it.
12. I was aware that I still had a lot of feelings about it, but I didn’t deal with them.
13. My feelings about it were kind of numb.
14. I found myself acting or feeling like I was back at that time.
15. I had trouble falling asleep.
16. I had waves of strong feelings about it.
17. I tried to remove it from my memory.
18. I had trouble concentrating.
19. Reminders of it caused me to have physical reactions, such as sweating, trouble breathing, nausea, or a pounding heart.
20. I had dreams about it.
21. I felt watchful and on-guard.
22. I tried not to talk about it.

**SI-3 Questionnaire for Assessment of Anxiety**

Anxiety Assessment (GAD-7 Questionnaire in Chinese) (1: no symptoms, 2: occasional, 3: usually; 4: always)

1. Feeling nervous, anxious, or on edge
2. Not being able to stop or control worrying
3. Worrying too much about different things
4. Trouble relaxing
5. Being so restless that it's hard to sit still
6. Becoming easily annoyed or irritable
7. Feeling afraid as if something awful might happen

**SI-4 Questionnaire for Assessment of Depression**

Depression Assessment (Center for Epidemiological Studies Depression (CES-D) questionnaire in Chinese) (1: no symptoms, 2: occasional, 3: usually; 4: always)

1. I was bothered by things that usually don’t bother me.
2. I did not feel like eating; my appetite was poor.
3. I felt that I could not shake off the blues even with help from my family.
4. I felt that I was just as good as other people.
5. I had trouble keeping my mind on what I was doing.
6. I felt depressed.
7. I felt that everything I did was an effort.
8. I did not feel hopeful about the future *.
9. I thought my life had been a failure.
10. I felt fearful.
11. My sleep was restless.
12. I was not happy *.
13. I talked less than usual.
14. I felt lonely.
15. People were unfriendly.
16. I did not enjoy life *.
17. I had crying spells.
18. I felt sad.
19. I felt that people disliked me.
20. I could not "get going."

Note: The questions marked with * were revised to be consistent with others as negative questions, and the original questions were (8) I felt hopeful about the future, (12) I was happy, (16) I enjoyed life. Such modification was made to avoid confusion according to the recommendation by Mingyuan Zhang and Yanling He.^48^

**SI-5 Questionnaire for Assessment of Mindfulness Practice Frequency**

Within the last two weeks, your practice situation regarding the exercises below corresponds to (1: no practice at all, 4: practice every second day, 7: daily practice)?

1. Body scan meditation
2. Open monitoring meditation
3. Zen nap
4. Focused-attention meditation to sounds
5. Focused-attention meditation to thoughts/ideas
6. Focused-attention meditation to speaking/listening
7. Focused-attention meditation to WeChat
8. Holistic focused-attention meditation
9. Pure mindfulness chanting
10. Pure mindfulness dancing
11. Compassion meditation

**Supplementary figure legends**

Supplementary figure 1. Age-dependence of depression and anxiety. Group-independent effect of age on self-reported symptoms of depression (estimated marginal means of normalized CES-D scores) and anxiety (estimated marginal means of normalized GAD-7 score) at peak time. * p < 0.05, ** p < 0.01, *** p <0.001. Error bars indicate the standard error. y = years.

**Supplementary table legends**

Supplementary table 1. Results of univariate analyses of variance (ANOVAs) for self-reported pandemic-related distress, depression, anxiety and stress in practitioners and non-practitioners at peak time. Bonferroni corrected threshold for statistical significance at p < 0.0125.

Supplementary table 2. Descriptive statistics.

Supplementary table 3. Results of hierarchical linear models (HLMs) for self-reported pandemic-related distress, depression, anxiety and stress at peak time and three-week follow-up in practitioners. Bonferroni corrected threshold for statistical significance at p < 0.0125.

Supplementary table 4. Linear models for the regression of improvement in self-reported pandemic-related distress, depression, anxiety and stress on practice frequency during the last two weeks, controlling for age, sex and baseline symptoms.

Supplementary Table 5. Linear models for the regression of improvement in self-reported pandemic-related distress, depression, anxiety and stress on practice frequency during the last two weeks, for the three subgroups of practitioners. Dis., pandemic-related distress; Dep., depression; Anx., anxiety; Str., stress; Beg., beginner; Int., intermediate; Adv., advanced.
