# Supplementary material for: Mindfulness practice for protecting mental health during the COVID-19 pandemic
Source: Transl Psychiatry. 2021 May 28;11:329. doi: 10.1038/s41398-021-01459-8 (PMC8160402; doi:10.1038/s41398-021-01459-8)
Supplement: Supplementary file 2 — Supplementary table 1 [file 41398_2021_1459_MOESM2_ESM.docx]

| **ANOVA Results** | | | | | | | |
| --- | --- | --- | --- | --- | --- | --- | --- |
|  | Source | Type III Sum of Squares | df | Mean Square | F | Sig. | Partial Eta Squared |
| Distress | Corrected Model | 15.464 | 27 | 0.573 | 14.873 | <0.001 | 0.155 |
|  | Intercept | 13.852 | 1 | 13.852 | 359.716 | <0.001 | 0.141 |
|  | Sex | 0.001 | 1 | 0.001 | 0.017 | 0.897 | <0.001 |
|  | Age | 0.338 | 4 | 0.084 | 2.191 | 0.068 | 0.004 |
|  | Group | 3.175 | 3 | 1.058 | 27.487 | <0.001 | 0.036 |
|  | Sex × Age | 0.090 | 4 | 0.023 | 0.585 | 0.673 | 0.001 |
|  | Sex × Group | 0.095 | 3 | 0.032 | 0.821 | 0.482 | 0.001 |
|  | Age × Group | 0.514 | 12 | 0.043 | 1.113 | 0.345 | 0.006 |
|  | Error | 84.523 | 2195 | 0.039 |  |  |  |
|  | Total | 379.101 | 2223 |  |  |  |  |
|  | Corrected Total | 99.987 | 2222 |  |  |  |  |
| Depression | Corrected Model | 1.33 | 27 | 0.049 | 3.081 | <0.001 | 0.037 |
|  | Intercept | 2.195 | 1 | 2.195 | 137.320 | <0.001 | 0.059 |
|  | Sex | 0.042 | 1 | 0.042 | 2.626 | 0.105 | 0.001 |
|  | Age | 0.378 | 4 | 0.095 | 5.912 | <0.001 | 0.011 |
|  | Group | 0.049 | 3 | 0.016 | 1.032 | 0.377 | 0.001 |
|  | Sex × Age | 0.054 | 4 | 0.014 | 0.849 | 0.494 | 0.002 |
|  | Sex × Group | 0.071 | 3 | 0.024 | 1.489 | 0.215 | 0.002 |
|  | Age × Group | 0.193 | 12 | 0.016 | 1.008 | 0.439 | 0.005 |
|  | Error | 35.094 | 2195 | 0.016 |  |  |  |
|  | Total | 69.177 | 2223 |  |  |  |  |
|  | Corrected Total | 36.424 | 2222 |  |  |  |  |
| Anxiety | Corrected Model | 1.592 | 27 | 0.059 | 2.675 | <0.001 | 0.032 |
|  | Intercept | 2.359 | 1 | 2.359 | 106.998 | <0.001 | 0.046 |
|  | Sex | 0.036 | 1 | 0.036 | 1.652 | 0.199 | 0.001 |
|  | Age | 0.569 | 4 | 0.142 | 6.455 | <0.001 | 0.012 |
|  | Group | 0.100 | 3 | 0.033 | 1.505 | 0.211 | 0.002 |
|  | Sex × Age | 0.051 | 4 | 0.013 | 0.583 | 0.675 | 0.001 |
|  | Sex × Group | 0.034 | 3 | 0.011 | 0.518 | 0.670 | 0.001 |
|  | Age × Group | 0.239 | 12 | 0.020 | 0.905 | 0.541 | 0.005 |
|  | Error | 48.394 | 2195 | 0.022 |  |  |  |
|  | Total | 87.585 | 2223 |  |  |  |  |
|  | Corrected Total | 49.986 | 2222 |  |  |  |  |
| Stress | Corrected Model | 0.556 | 27 | 0.021 | 1.495 | 0.049 | 0.018 |
|  | Intercept | 2.826 | 1 | 2.826 | 205.338 | <0.001 | 0.086 |
|  | Sex | <0.001 | 1 | <0.001 | 0.016 | 0.900 | <0.001 |
|  | Age | 0.057 | 4 | 0.014 | 1.040 | 0.385 | 0.002 |
|  | Group | 0.082 | 3 | 0.027 | 1.979 | 0.115 | 0.003 |
|  | Sex × Age | 0.064 | 4 | 0.016 | 1.167 | 0.323 | 0.002 |
|  | Sex × Group | 0.013 | 3 | 0.004 | 0.325 | 0.807 | <0.001 |
|  | Age × Group | 0.088 | 12 | 0.007 | 0.532 | 0.895 | 0.003 |
|  | Error | 30.211 | 2195 | 0.014 |  |  |  |
|  | Total | 66.933 | 2223 |  |  |  |  |
|  | Corrected Total | 30.766 | 2222 |  |  |  |  |

Supplementary table 1. Results of univariate analyses of variance (ANOVAs) for self-reported pandemic-related distress, depression, anxiety and stress in practitioners and non-practitioners at peak time. Bonferroni corrected threshold for statistical significance at p < 0.0125.
