# Supplementary material for: Mindfulness practice for protecting mental health during the COVID-19 pandemic
Source: Transl Psychiatry. 2021 May 28;11:329. doi: 10.1038/s41398-021-01459-8 (PMC8160402; doi:10.1038/s41398-021-01459-8)
Supplement: Supplementary file 3 — Supplementary table 2 [file 41398_2021_1459_MOESM3_ESM.docx]

|  | Group | N | Mean | Std. deviation | Mean of raw scores | Std. deviation of raw scores |
| --- | --- | --- | --- | --- | --- | --- |
| Distress | Non-practitioners | 1550 | 0.405 | 0.207 | 17.15 | 6.20 |
|  | Practitioners: peak | 673 | 0.237 | 0.176 | 12.12 | 5.27 |
|  | Practitioners: 3wk | 521 | 0.165 | 0.145 | 9.95 | 4.34 |
| Depression | Non-practitioners | 1550 | 0.122 | 0.128 | 27.30 | 7.69 |
|  | Practitioners: peak | 673 | 0.121 | 0.128 | 27.24 | 7.66 |
|  | Practitioners: 3wk | 521 | 0.145 | 0.138 | 28.71 | 8.28 |
| Anxiety | Non-practitioners | 1550 | 0.131 | 0.152 | 10.66 | 4.25 |
|  | Practitioners: peak | 673 | 0.128 | 0.146 | 10.59 | 4.09 |
|  | Practitioners: 3wk | 521 | 0.122 | 0.126 | 10.40 | 3.52 |
| Stress | Non-practitioners | 1550 | 0.131 | 0.121 | 19.68 | 7.96 |
|  | Practitioners: peak | 673 | 0.119 | 0.110 | 18.83 | 7.28 |
|  | Practitioners: 3wk | 521 | 0.117 | 0.111 | 18.72 | 7.32 |

Supplementary table 2. Descriptive statistics.
