# Supplementary material for: Mindfulness practice for protecting mental health during the COVID-19 pandemic
Source: Transl Psychiatry. 2021 May 28;11:329. doi: 10.1038/s41398-021-01459-8 (PMC8160402; doi:10.1038/s41398-021-01459-8)
Supplement: Supplementary file 4 — Supplementary table 3 [file 41398_2021_1459_MOESM4_ESM.docx]

| **HLM Results** | | | | | |
| --- | --- | --- | --- | --- | --- |
|  | Source | Numerator df | Denominator df | F | Sig. |
| Distress | Intercept | 1 | 864.272 | 76.355 | <0.001 |
|  | Time | 1 | 616.121 | 6.848 | 0.009 |
|  | Experience | 2 | 887.231 | 1.382 | 0.252 |
|  | Age | 4 | 962.466 | 1.035 | 0.388 |
|  | Sex | 1 | 843.912 | 0.624 | 0.430 |
|  | Practice Freq. | 1 | 938.235 | 1.478 | 0.224 |
|  | Time × Experience | 2 | 569.317 | 2.360 | 0.095 |
|  | Time × Age | 4 | 546.106 | 4.007 | 0.003 |
|  | Time × Sex | 1 | 571.373 | 0.492 | 0.483 |
|  | Experience × Age | 8 | 706.030 | 1.654 | 0.106 |
|  | Experience × Sex | 2 | 761.225 | 1.504 | 0.223 |
|  | Age × Sex | 4 | 650.084 | 1.566 | 0.182 |
|  | Time × Practice Freq. | 1 | 640.760 | 0.418 | 0.518 |
|  | Experience × Practice Freq. | 2 | 1118.551 | 1.792 | 0.167 |
|  | Age × Practice Freq. | 4 | 1022.489 | 1.587 | 0.176 |
|  | Sex × Practice Freq. | 1 | 1097.083 | 0.402 | 0.526 |
| Depression | Intercept | 1 | 973.197 | 87.424 | <0.001 |
|  | Time | 1 | 546.766 | 20.353 | <0.001 |
|  | Experience | 2 | 961.859 | 0.057 | 0.945 |
|  | Age | 4 | 1024.340 | 1.851 | 0.117 |
|  | Sex | 1 | 895.494 | 0.451 | 0.502 |
|  | Practice Freq. | 1 | 1060.453 | 12.527 | <0.001 |
|  | Time × Experience | 2 | 517.365 | 1.152 | 0.317 |
|  | Time × Age | 4 | 503.962 | 0.973 | 0.422 |
|  | Time × Sex | 1 | 511.482 | 1.175 | 0.279 |
|  | Experience × Age | 8 | 760.235 | 0.845 | 0.563 |
|  | Experience × Sex | 2 | 763.689 | 3.487 | 0.031 |
|  | Age × Sex | 4 | 696.876 | 0.861 | 0.487 |
|  | Time × Practice Freq. | 1 | 563.372 | 5.937 | 0.015 |
|  | Experience × Practice Freq. | 2 | 1116.222 | 0.384 | 0.681 |
|  | Age × Practice Freq. | 4 | 1075.395 | 0.234 | 0.919 |
|  | Sex × Practice Freq. | 1 | 1076.878 | 1.516 | 0.218 |
| Anxiety | Intercept | 1 | 848.086 | 83.368 | <0.001 |
|  | Time | 1 | 610.884 | 2.597 | 0.108 |
|  | Experience | 2 | 885.060 | 0.312 | 0.732 |
|  | Age | 4 | 948.160 | 3.370 | 0.009 |
|  | Sex | 1 | 836.951 | 0.006 | 0.937 |
|  | Practice Freq. | 1 | 921.902 | 15.103 | <0.001 |
|  | Time × Experience | 2 | 564.865 | 0.656 | 0.519 |
|  | Time × Age | 4 | 541.772 | 1.527 | 0.193 |
|  | Time × Sex | 1 | 564.036 | 0.757 | 0.385 |
|  | Experience × Age | 8 | 706.819 | 0.332 | 0.954 |
|  | Experience × Sex | 2 | 754.418 | 3.522 | 0.030 |
|  | Age × Sex | 4 | 649.563 | 0.893 | 0.468 |
|  | Time × Practice Freq. | 1 | 637.842 | 0.995 | 0.319 |
|  | Experience × Practice Freq. | 2 | 1103.278 | 1.992 | 0.137 |
|  | Age × Practice Freq. | 4 | 1008.490 | 1.277 | 0.277 |
|  | Sex × Practice Freq. | 1 | 1075.838 | 0.351 | 0.554 |
| Stress | Intercept | 1 | 919.269 | 82.804 | <0.001 |
|  | Time | 1 | 579.919 | 4.999 | 0.026 |
|  | Experience | 2 | 934.802 | 0.600 | 0.549 |
|  | Age | 4 | 996.377 | 0.445 | 0.776 |
|  | Sex | 1 | 875.803 | 5.702 | 0.017 |
|  | Practice Freq. | 1 | 1002.031 | 6.577 | 0.010 |
|  | Time × Experience | 2 | 542.725 | 0.095 | 0.910 |
|  | Time × Age | 4 | 524.692 | 1.162 | 0.327 |
|  | Time × Sex | 1 | 537.926 | 1.431 | 0.232 |
|  | Experience × Age | 8 | 747.490 | 0.838 | 0.569 |
|  | Experience × Sex | 2 | 769.271 | 0.124 | 0.883 |
|  | Age × Sex | 4 | 684.782 | 0.540 | 0.707 |
|  | Time × Practice Freq. | 1 | 601.435 | 3.259 | 0.072 |
|  | Experience × Practice Freq. | 2 | 1119.623 | 0.467 | 0.627 |
|  | Age × Practice Freq. | 4 | 1055.399 | 0.271 | 0.897 |
|  | Sex × Practice Freq. | 1 | 1080.377 | 3.342 | 0.068 |

Supplementary table 3. Results of hierarchical linear models (HLMs) for self-reported pandemic-related distress, depression, anxiety and stress at peak time and three-week follow-up in practitioners. Bonferroni corrected threshold for statistical significance at p < 0.0125.
