# Supplementary material for: Mindfulness practice for protecting mental health during the COVID-19 pandemic
Source: Transl Psychiatry. 2021 May 28;11:329. doi: 10.1038/s41398-021-01459-8 (PMC8160402; doi:10.1038/s41398-021-01459-8)
Supplement: Supplementary file 5 — Supplementary table 4 [file 41398_2021_1459_MOESM5_ESM.docx]

| **Regression Results** | | | | | | | | | | | |
| --- | --- | --- | --- | --- | --- | --- | --- | --- | --- | --- | --- |
|  |  |  |  | 95,0% Confidence Interval for B | |  |  |  | Correlations | | |
|  |  | B | Std. Error | Lower Bound | Upper Bound | β | t | Sig. | Zero-order | Partial | Semi-  partial |
| Distress | (Constant) | -0.150 | 0.038 | -0.225 | -0.075 |  | -3.927 | 0.000 |  |  |  |
|  | Practice Frequency | 0.026 | 0.026 | -0.024 | 0.076 | 0.039 | 1.009 | 0.313 | -0.096 | 0.048 | 0.037 |
|  | 25-30 | 0.116 | 0.042 | 0.034 | 0.199 | 0.162 | 2.767 | 0.006 | 0.057 | 0.131 | 0.103 |
|  | 31-40 | 0.095 | 0.034 | 0.029 | 0.162 | 0.293 | 2.803 | 0.005 | 0.132 | 0.133 | 0.104 |
|  | 41-50 | 0.068 | 0.034 | 0.002 | 0.134 | 0.217 | 2.018 | 0.044 | -0.045 | 0.096 | 0.075 |
|  | 51-60 | 0.046 | 0.036 | -0.024 | 0.117 | 0.103 | 1.293 | 0.197 | -0.084 | 0.062 | 0.048 |
|  | Sex | -0.015 | 0.014 | -0.042 | 0.012 | -0.042 | -1.116 | 0.265 | -0.050 | -0.053 | -0.041 |
|  | Baseline Distress | 0.564 | 0.035 | 0.495 | 0.633 | 0.606 | 16.008 | 0.000 | 0.610 | 0.608 | 0.594 |
| F(7,437) = 41.274, p < 0.001, R^2 = 0.398 | | | | | | | | | | | |
| Depression | (Constant) | -0.140 | 0.032 | -0.203 | -0.078 |  | -4.391 | 0.000 |  |  |  |
|  | Practice Frequency | 0.108 | 0.022 | 0.066 | 0.151 | 0.225 | 5.003 | 0.000 | 0.118 | 0.233 | 0.214 |
|  | 25-30 | -0.017 | 0.035 | -0.086 | 0.052 | -0.032 | -0.476 | 0.634 | -0.021 | -0.023 | -0.020 |
|  | 31-40 | 0.010 | 0.028 | -0.046 | 0.066 | 0.041 | 0.342 | 0.732 | 0.024 | 0.016 | 0.015 |
|  | 41-50 | 0.018 | 0.028 | -0.038 | 0.073 | 0.077 | 0.623 | 0.533 | 0.036 | 0.030 | 0.027 |
|  | 51-60 | -0.011 | 0.030 | -0.070 | 0.048 | -0.034 | -0.375 | 0.708 | -0.066 | -0.018 | -0.016 |
|  | Sex | -0.006 | 0.011 | -0.028 | 0.016 | -0.023 | -0.525 | 0.600 | -0.060 | -0.025 | -0.022 |
|  | Baseline Depression | 0.403 | 0.041 | 0.322 | 0.484 | 0.436 | 9.804 | 0.000 | 0.379 | 0.425 | 0.420 |
| F(7,437) = 15.586, p < 0.001, R^2 = 0.200 | | | | | | | | | | | |
| Anxiety | (Constant) | -0.157 | 0.032 | -0.219 | -0.095 |  | -4.968 | 0.000 |  |  |  |
|  | Practice Frequency | 0.095 | 0.022 | 0.052 | 0.137 | 0.171 | 4.374 | 0.000 | -0.005 | 0.205 | 0.163 |
|  | 25-30 | 0.012 | 0.035 | -0.057 | 0.081 | 0.019 | 0.332 | 0.740 | -0.020 | 0.016 | 0.012 |
|  | 31-40 | 0.038 | 0.028 | -0.018 | 0.094 | 0.141 | 1.347 | 0.179 | 0.062 | 0.064 | 0.050 |
|  | 41-50 | 0.039 | 0.028 | -0.017 | 0.094 | 0.148 | 1.373 | 0.170 | -0.004 | 0.066 | 0.051 |
|  | 51-60 | 0.030 | 0.030 | -0.029 | 0.090 | 0.081 | 1.013 | 0.312 | -0.017 | 0.048 | 0.038 |
|  | Sex | 0.005 | 0.011 | -0.017 | 0.027 | 0.017 | 0.440 | 0.660 | -0.037 | 0.021 | 0.016 |
|  | Baseline Anxiety | 0.601 | 0.036 | 0.530 | 0.672 | 0.646 | 16.624 | 0.000 | 0.605 | 0.622 | 0.618 |
| F(7,437) = 40.825, p < 0.001, R^2 = 0.395 | | | | | | | | | | | |
| Stress | (Constant) | -0.109 | 0.029 | -0.165 | -0.053 |  | -3.806 | 0.000 |  |  |  |
|  | Practice Frequency | 0.061 | 0.019 | 0.023 | 0.098 | 0.139 | 3.176 | 0.002 | 0.033 | 0.150 | 0.134 |
|  | 25-30 | 0.010 | 0.031 | -0.052 | 0.071 | 0.021 | 0.310 | 0.757 | -0.030 | 0.015 | 0.013 |
|  | 31-40 | 0.033 | 0.025 | -0.017 | 0.082 | 0.153 | 1.289 | 0.198 | 0.066 | 0.062 | 0.054 |
|  | 41-50 | 0.024 | 0.025 | -0.025 | 0.073 | 0.116 | 0.954 | 0.341 | -0.007 | 0.046 | 0.040 |
|  | 51-60 | 0.008 | 0.027 | -0.045 | 0.060 | 0.026 | 0.294 | 0.769 | -0.041 | 0.014 | 0.012 |
|  | Sex | -0.007 | 0.010 | -0.027 | 0.013 | -0.029 | -0.685 | 0.494 | -0.045 | -0.033 | -0.029 |
|  | Baseline Stress | 0.452 | 0.041 | 0.371 | 0.533 | 0.472 | 10.972 | 0.000 | 0.446 | 0.465 | 0.462 |
| F(7,437) = 18.024, p < 0.001, R^2 = 0.212 | | | | | | | | | | | |

Supplementary table 4. Linear models for the regression of improvement in self-reported pandemic-related distress, depression, anxiety and stress on practice frequency during the last two weeks, controlling for age, sex and baseline symptoms.
