# Supplementary material for: Mindfulness practice for protecting mental health during the COVID-19 pandemic
Source: Transl Psychiatry. 2021 May 28;11:329. doi: 10.1038/s41398-021-01459-8 (PMC8160402; doi:10.1038/s41398-021-01459-8)
Supplement: Supplementary file 6 — Supplementary table 5 [file 41398_2021_1459_MOESM6_ESM.docx]

|  | **Regression Results** | | | | | | | | | | | |
| --- | --- | --- | --- | --- | --- | --- | --- | --- | --- | --- | --- | --- |
|  |  |  |  |  | 95,0% Confidence Interval for B | |  |  |  | Correlations | | |
|  |  |  | B | Std. Error | Lower Bound | Upper Bound | β | t | Sig. | Zero-order | Partial | Part |
| Dis. | Beg. | (Constant) | -0.329 | 0.080 | -0.487 | -0.171 |  | -4.122 | <0.001 |  |  |  |
|  |  | Practice Frequency | 0.063 | 0.051 | -0.039 | 0.164 | 0.078 | 1.215 | 0.226 | -0.057 | 0.103 | 0.074 |
|  |  | 25-30 | 0.286 | 0.108 | 0.073 | 0.500 | 0.235 | 2.656 | 0.009 | 0.071 | 0.220 | 0.162 |
|  |  | 31-40 | 0.249 | 0.078 | 0.094 | 0.404 | 0.699 | 3.181 | 0.002 | 0.187 | 0.261 | 0.194 |
|  |  | 41-50 | 0.226 | 0.078 | 0.071 | 0.381 | 0.648 | 2.887 | 0.005 | -0.054 | 0.239 | 0.176 |
|  |  | 51-60 | 0.226 | 0.079 | 0.071 | 0.382 | 0.501 | 2.875 | 0.005 | -0.064 | 0.238 | 0.175 |
|  |  | Sex | -0.021 | 0.025 | -0.071 | 0.029 | -0.053 | -0.829 | 0.408 | -0.059 | -0.070 | -0.051 |
|  |  | Baseline Distress | 0.588 | 0.062 | 0.466 | 0.710 | 0.620 | 9.517 | <0.001 | 0.657 | 0.629 | 0.581 |
|  | F(7,138) = 18.664, p < 0.001, R^2 = 0.486 | | | | | | | | | | | |
|  | Int. | (Constant) | -0.091 | 0.085 | -0.260 | 0.079 |  | -1.066 | 0.290 |  |  |  |
|  |  | Practice Frequency | 0.067 | 0.060 | -0.052 | 0.185 | 0.094 | 1.120 | 0.266 | -0.093 | 0.123 | 0.087 |
|  |  | 25-30 | 0.057 | 0.083 | -0.108 | 0.222 | 0.095 | 0.690 | 0.492 | 0.093 | 0.076 | 0.053 |
|  |  | 31-40 | -0.009 | 0.073 | -0.154 | 0.137 | -0.026 | -0.118 | 0.907 | 0.058 | -0.013 | -0.009 |
|  |  | 41-50 | -0.043 | 0.073 | -0.188 | 0.102 | -0.128 | -0.588 | 0.558 | -0.096 | -0.065 | -0.046 |
|  |  | 51-60 | -0.095 | 0.081 | -0.257 | 0.066 | -0.168 | -1.174 | 0.244 | -0.061 | -0.129 | -0.091 |
|  |  | Sex | <0.001 | 0.034 | -0.068 | 0.068 | <0.001 | 0.001 | 0.999 | -0.042 | <0.001 | <0.001 |
|  |  | Baseline Distress | 0.667 | 0.075 | 0.519 | 0.815 | 0.725 | 8.949 | <0.001 | 0.680 | 0.705 | 0.693 |
|  | F(7,81) = 12.242, p < 0.001, R^2 = 0.514 | | | | | | | | | | | |
|  | Adv. | (Constant) | -0.088 | 0.053 | -0.193 | 0.017 |  | -1.647 | 0.101 |  |  |  |
|  |  | Practice Frequency | -0.008 | 0.039 | -0.085 | 0.069 | -0.013 | -0.210 | 0.834 | -0.077 | -0.015 | -0.012 |
|  |  | 25-30 | 0.064 | 0.055 | -0.044 | 0.172 | 0.107 | 1.173 | 0.242 | 0.042 | 0.082 | 0.069 |
|  |  | 31-40 | 0.072 | 0.044 | -0.016 | 0.160 | 0.241 | 1.622 | 0.106 | 0.108 | 0.113 | 0.096 |
|  |  | 41-50 | 0.045 | 0.044 | -0.041 | 0.131 | 0.161 | 1.025 | 0.307 | -0.003 | 0.072 | 0.061 |
|  |  | 51-60 | 0.008 | 0.048 | -0.086 | 0.103 | 0.020 | 0.173 | 0.863 | -0.121 | 0.012 | 0.010 |
|  |  | Sex | -0.010 | 0.018 | -0.046 | 0.027 | -0.031 | -0.517 | 0.606 | -0.035 | -0.036 | -0.031 |
|  |  | Baseline Distress | 0.471 | 0.055 | 0.363 | 0.579 | 0.511 | 8.598 | <0.001 | 0.514 | 0.518 | 0.509 |
|  | F(7,202) = 11.929, p < 0.001, R^2 = 0.292 | | | | | | | | | | | |
| Dep. | Beg. | (Constant) | -0.194 | 0.076 | -0.345 | -0.043 |  | -2.541 | 0.012 |  |  |  |
|  |  | Practice Frequency | 0.093 | 0.049 | -0.003 | 0.189 | 0.159 | 1.920 | 0.057 | 0.114 | 0.161 | 0.154 |
|  |  | 25-30 | 0.038 | 0.101 | -0.162 | 0.238 | 0.043 | 0.377 | 0.707 | -0.030 | 0.032 | 0.030 |
|  |  | 31-40 | 0.077 | 0.073 | -0.067 | 0.221 | 0.296 | 1.054 | 0.294 | 0.070 | 0.089 | 0.084 |
|  |  | 41-50 | 0.071 | 0.073 | -0.073 | 0.215 | 0.280 | 0.974 | 0.332 | 0.012 | 0.083 | 0.078 |
|  |  | 51-60 | 0.054 | 0.074 | -0.092 | 0.200 | 0.165 | 0.732 | 0.465 | -0.052 | 0.062 | 0.059 |
|  |  | Sex | 0.002 | 0.024 | -0.045 | 0.050 | 0.008 | 0.098 | 0.922 | 0.011 | 0.008 | 0.008 |
|  |  | Baseline Depression | 0.295 | 0.084 | 0.129 | 0.461 | 0.292 | 3.521 | 0.001 | 0.283 | 0.287 | 0.282 |
|  | F(7,138) = 2.556, p = 0.017, R^2 = 0.115 | | | | | | | | | | | |
|  | Int. | (Constant) | -0.086 | 0.062 | -0.210 | 0.037 |  | -1.388 | 0.169 |  |  |  |
|  |  | Practice Frequency | 0.122 | 0.045 | 0.033 | 0.212 | 0.272 | 2.724 | 0.008 | 0.156 | 0.290 | 0.255 |
|  |  | 25-30 | -0.080 | 0.063 | -0.206 | 0.046 | -0.210 | -1.261 | 0.211 | 0.004 | -0.139 | -0.118 |
|  |  | 31-40 | -0.036 | 0.056 | -0.147 | 0.075 | -0.171 | -0.639 | 0.525 | 0.071 | -0.071 | -0.060 |
|  |  | 41-50 | -0.047 | 0.056 | -0.158 | 0.063 | -0.223 | -0.851 | 0.397 | -0.095 | -0.094 | -0.080 |
|  |  | 51-60 | -0.077 | 0.062 | -0.202 | 0.047 | -0.215 | -1.241 | 0.218 | -0.027 | -0.137 | -0.116 |
|  |  | Sex | 0.010 | 0.027 | -0.043 | 0.063 | 0.037 | 0.382 | 0.704 | -0.086 | 0.042 | 0.036 |
|  |  | Baseline Depression | 0.417 | 0.079 | 0.259 | 0.574 | 0.527 | 5.270 | <0.001 | 0.449 | 0.505 | 0.494 |
|  | F(7,81) = 4.721, p < 0.001, R^2 = 0.290 | | | | | | | | | | | |
|  | Adv. | (Constant) | -0.151 | 0.042 | -0.235 | -0.068 |  | -3.573 | <0.001 |  |  |  |
|  |  | Practice Frequency | 0.105 | 0.032 | 0.043 | 0.168 | 0.211 | 3.316 | 0.001 | 0.059 | 0.227 | 0.198 |
|  |  | 25-30 | -0.011 | 0.043 | -0.095 | 0.073 | -0.023 | -0.250 | 0.803 | -0.051 | -0.018 | -0.015 |
|  |  | 31-40 | 0.008 | 0.035 | -0.060 | 0.076 | 0.034 | 0.228 | 0.820 | -0.029 | 0.016 | 0.014 |
|  |  | 41-50 | 0.033 | 0.034 | -0.034 | 0.100 | 0.154 | 0.970 | 0.333 | 0.106 | 0.068 | 0.058 |
|  |  | 51-60 | -0.002 | 0.037 | -0.075 | 0.072 | -0.005 | -0.041 | 0.968 | -0.075 | -0.003 | -0.002 |
|  |  | Sex | -0.007 | 0.015 | -0.036 | 0.022 | -0.031 | -0.495 | 0.621 | -0.106 | -0.035 | -0.030 |
|  |  | Baseline Depression | 0.484 | 0.059 | 0.369 | 0.600 | 0.524 | 8.263 | <0.001 | 0.466 | 0.503 | 0.494 |
|  | F(7,202) = 11.050, p < 0.001, R^2 = 0.277 | | | | | | | | | | | |
| Anx. | Beg. | (Constant) | -0.244 | 0.070 | -0.384 | -0.105 |  | -3.465 | 0.001 |  |  |  |
|  |  | Practice Frequency | 0.065 | 0.045 | -0.023 | 0.154 | 0.095 | 1.455 | 0.148 | -0.002 | 0.123 | 0.092 |
|  |  | 25-30 | 0.107 | 0.094 | -0.079 | 0.292 | 0.103 | 1.138 | 0.257 | -0.023 | 0.096 | 0.072 |
|  |  | 31-40 | 0.144 | 0.068 | 0.009 | 0.279 | 0.472 | 2.114 | 0.036 | 0.184 | 0.177 | 0.133 |
|  |  | 41-50 | 0.126 | 0.068 | -0.009 | 0.260 | 0.422 | 1.850 | 0.067 | -0.087 | 0.156 | 0.116 |
|  |  | 51-60 | 0.124 | 0.069 | -0.012 | 0.261 | 0.322 | 1.804 | 0.073 | -0.016 | 0.152 | 0.114 |
|  |  | Sex | -0.020 | 0.022 | -0.064 | 0.024 | -0.058 | -0.895 | 0.372 | -0.082 | -0.076 | -0.056 |
|  |  | Baseline Anxiety | 0.594 | 0.062 | 0.472 | 0.716 | 0.629 | 9.620 | <0.001 | 0.647 | 0.634 | 0.605 |
|  | F(7,138) = 16.363, p < 0.001, R^2 = 0.454 | | | | | | | | | | | |
|  | Int. | (Constant) | -0.063 | 0.077 | -0.215 | 0.090 |  | -0.816 | 0.417 |  |  |  |
|  |  | Practice Frequency | 0.047 | 0.057 | -0.065 | 0.160 | 0.076 | 0.834 | 0.407 | -0.134 | 0.092 | 0.070 |
|  |  | 25-30 | -0.056 | 0.077 | -0.210 | 0.098 | -0.107 | -0.723 | 0.472 | 0.013 | -0.080 | -0.061 |
|  |  | 31-40 | -0.037 | 0.068 | -0.173 | 0.100 | -0.128 | -0.536 | 0.594 | 0.086 | -0.059 | -0.045 |
|  |  | 41-50 | -0.051 | 0.068 | -0.186 | 0.085 | -0.175 | -0.744 | 0.459 | -0.114 | -0.082 | -0.062 |
|  |  | 51-60 | -0.048 | 0.076 | -0.199 | 0.104 | -0.096 | -0.623 | 0.535 | 0.020 | -0.069 | -0.052 |
|  |  | Sex | 0.019 | 0.032 | -0.045 | 0.082 | 0.049 | 0.578 | 0.565 | 0.010 | 0.064 | 0.048 |
|  |  | Baseline Anxiety | 0.645 | 0.085 | 0.476 | 0.815 | 0.673 | 7.560 | <0.001 | 0.648 | 0.643 | 0.633 |
|  | F(7,81) = 8.784, p < 0.001, R^2 = 0.432 | | | | | | | | | | | |
|  | Adv. | (Constant) | -0.153 | 0.040 | -0.232 | -0.074 |  | -3.832 | <0.001 |  |  |  |
|  |  | Practice Frequency | 0.108 | 0.030 | 0.049 | 0.168 | 0.211 | 3.596 | <0.001 | 0.056 | 0.245 | 0.201 |
|  |  | 25-30 | -0.001 | 0.041 | -0.082 | 0.080 | -0.003 | -0.029 | 0.977 | -0.048 | -0.002 | -0.002 |
|  |  | 31-40 | 0.018 | 0.033 | -0.048 | 0.084 | 0.075 | 0.538 | 0.591 | -0.072 | 0.038 | 0.030 |
|  |  | 41-50 | 0.043 | 0.033 | -0.022 | 0.107 | 0.194 | 1.302 | 0.195 | 0.133 | 0.091 | 0.073 |
|  |  | 51-60 | 0.026 | 0.036 | -0.045 | 0.097 | 0.080 | 0.729 | 0.467 | -0.031 | 0.051 | 0.041 |
|  |  | Sex | 0.017 | 0.014 | -0.011 | 0.045 | 0.071 | 1.225 | 0.222 | -0.017 | 0.086 | 0.069 |
|  |  | Baseline Anxiety | 0.570 | 0.054 | 0.463 | 0.677 | 0.612 | 10.460 | <0.001 | 0.553 | 0.593 | 0.585 |
|  | F(7,202) = 16.763, p < 0.001, R^2 = 0.367 | | | | | | | | | | | |
| Str. | Beg. | (Constant) | -0.168 | 0.062 | -0.290 | -0.046 |  | -2.730 | 0.007 |  |  |  |
|  |  | Practice Frequency | 0.079 | 0.039 | 0.002 | 0.156 | 0.151 | 2.028 | 0.044 | 0.107 | 0.170 | 0.146 |
|  |  | 25-30 | 0.057 | 0.081 | -0.103 | 0.217 | 0.072 | 0.701 | 0.484 | -0.033 | 0.060 | 0.051 |
|  |  | 31-40 | 0.082 | 0.058 | -0.033 | 0.197 | 0.353 | 1.402 | 0.163 | 0.120 | 0.119 | 0.101 |
|  |  | 41-50 | 0.063 | 0.059 | -0.052 | 0.179 | 0.280 | 1.082 | 0.281 | -0.039 | 0.092 | 0.078 |
|  |  | 51-60 | 0.043 | 0.059 | -0.074 | 0.160 | 0.147 | 0.724 | 0.470 | -0.044 | 0.062 | 0.052 |
|  |  | Sex | -0.005 | 0.019 | -0.043 | 0.033 | -0.019 | -0.257 | 0.797 | -0.017 | -0.022 | -0.019 |
|  |  | Baseline Stress | 0.437 | 0.065 | 0.309 | 0.565 | 0.494 | 6.730 | <0.001 | 0.488 | 0.497 | 0.486 |
|  | F(7,138) = 7.671, p < 0.001, R^2 = 0.280 | | | | | | | | | | | |
|  | Int. | (Constant) | -0.108 | 0.064 | -0.236 | 0.020 |  | -1.680 | 0.097 |  |  |  |
|  |  | Practice Frequency | 0.016 | 0.046 | -0.076 | 0.108 | 0.036 | 0.355 | 0.724 | -0.118 | 0.039 | 0.033 |
|  |  | 25-30 | 0.014 | 0.064 | -0.114 | 0.141 | 0.035 | 0.212 | 0.832 | -0.114 | 0.024 | 0.020 |
|  |  | 31-40 | 0.047 | 0.057 | -0.066 | 0.160 | 0.221 | 0.830 | 0.409 | 0.106 | 0.092 | 0.077 |
|  |  | 41-50 | 0.045 | 0.056 | -0.068 | 0.157 | 0.207 | 0.794 | 0.430 | -0.009 | 0.088 | 0.074 |
|  |  | 51-60 | 0.028 | 0.063 | -0.098 | 0.154 | 0.076 | 0.443 | 0.659 | -0.011 | 0.049 | 0.041 |
|  |  | Sex | -0.038 | 0.027 | -0.091 | 0.015 | -0.136 | -1.435 | 0.155 | -0.126 | -0.158 | -0.134 |
|  |  | Baseline Stress | 0.582 | 0.109 | 0.364 | 0.800 | 0.518 | 5.317 | <0.001 | 0.515 | 0.509 | 0.495 |
|  | F(7,81) = 4.884, p < 0.001, R^2 = 0.297 | | | | | | | | | | | |
|  | Adv. | (Constant) | -0.077 | 0.039 | -0.155 | <0.001 |  | -1.963 | 0.051 |  |  |  |
|  |  | Practice Frequency | 0.047 | 0.029 | -0.010 | 0.104 | 0.109 | 1.641 | 0.102 | 0.029 | 0.115 | 0.105 |
|  |  | 25-30 | -0.004 | 0.040 | -0.083 | 0.074 | -0.010 | -0.106 | 0.916 | 0.006 | -0.007 | -0.007 |
|  |  | 31-40 | 0.009 | 0.032 | -0.055 | 0.073 | 0.046 | 0.286 | 0.776 | <0.001 | 0.020 | 0.018 |
|  |  | 41-50 | 0.006 | 0.032 | -0.057 | 0.068 | 0.030 | 0.174 | 0.862 | 0.021 | 0.012 | 0.011 |
|  |  | 51-60 | <0.001 | 0.035 | -0.069 | 0.069 | -0.001 | -0.009 | 0.993 | -0.043 | -0.001 | -0.001 |
|  |  | Sex | 0.001 | 0.014 | -0.025 | 0.028 | 0.007 | 0.109 | 0.913 | -0.035 | 0.008 | 0.007 |
|  |  | Baseline Stress | 0.429 | 0.066 | 0.298 | 0.560 | 0.425 | 6.455 | <0.001 | 0.403 | 0.414 | 0.413 |
|  | F(7,202) = 6.109, p < 0.001, R^2 = 0.175 | | | | | | | | | | | |

Supplementary Table 5. Linear models for the regression of improvement in self-reported pandemic-related distress, depression, anxiety and stress on practice frequency during the last two weeks, for the three subgroups of practitioners. Dis., pandemic-related distress; Dep., depression; Anx., anxiety; Str., stress; Beg., beginner; Int., intermediate; Adv., advanced.
