# Supplementary figures and images for: Mindfulness practice for protecting mental health during the COVID-19 pandemic
Source: Transl Psychiatry. 2021 May 28;11:329. doi: 10.1038/s41398-021-01459-8 (PMC8160402; doi:10.1038/s41398-021-01459-8)

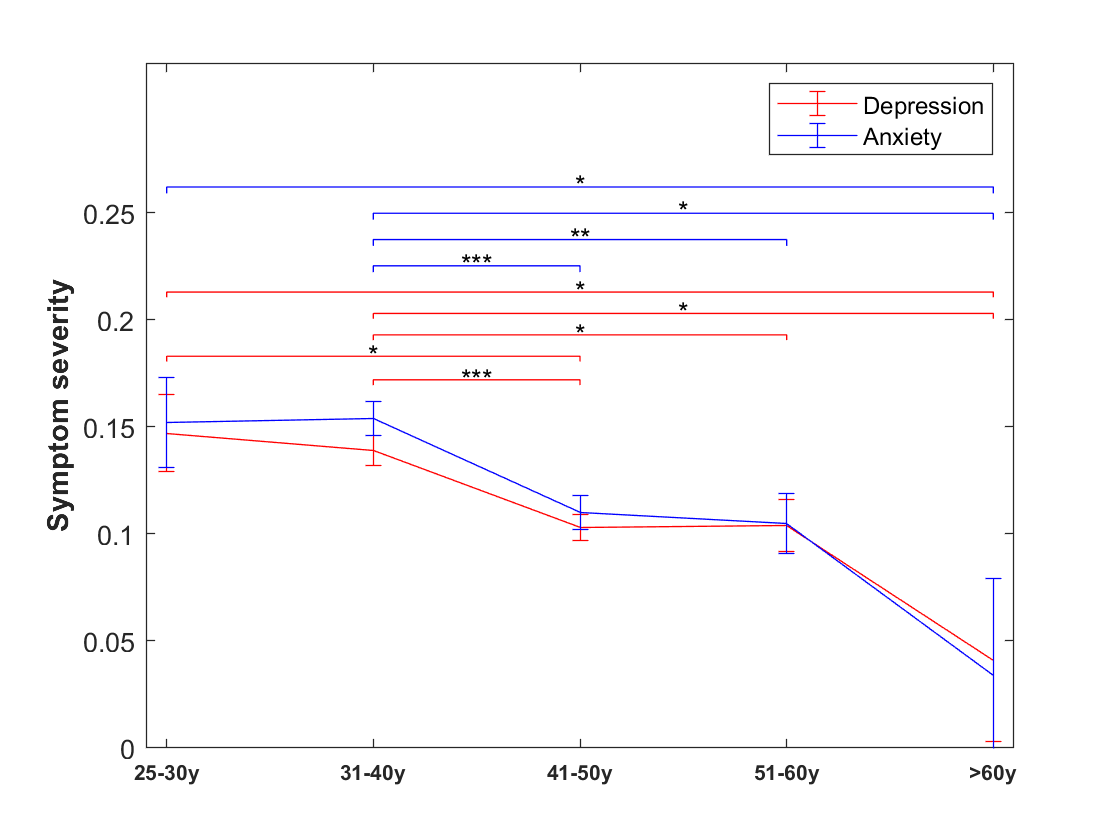

Supplement: Supplementary file 7 — Supplementary figure 1 [file 41398_2021_1459_MOESM7_ESM.tif]
